# Endoscopic repair of duodenal fistula occurring as a rare complication of abdominal drainage following partial hepatectomy

**DOI:** 10.1055/a-2268-5793

**Published:** 2024-03-11

**Authors:** Ruide Liu, Xianglei Yuan, Xinyue Zhou, Qianyi Deng, Bing Hu

**Affiliations:** 1 34753 Department of Gastroenterology and Hepatology/Medical Engineering Integration Laboratory of Digestive Endoscopy, West China Hospital of Sichuan University, Chengdu, China

The placement of abdominal drainage tubes is a standard procedure following abdominal surgery 1 2 . Here, we present a case of duodenal fistula caused by drainage tubes following partial hepatectomy.

A 54-year-old man was referred to our hospital due to a 3-day history of incision pain and increased drainage. Sixteen days earlier, he had undergone partial hepatectomy and cholecystectomy for hepatocellular carcinoma, with two drainage tubes placed. No intraoperative or postoperative complications were noted, and the patient was discharged on postoperative day 7. Upon examination, his vital signs were stable. Abdominal enhanced CT showed localized thinning of the duodenal bulb wall, closely adjacent to one of the drainage tubes ( Fig. 1 **a** ). Endoscopy demonstrated a 0.8-cm-diameter fistula in the duodenal bulb, with drainage tubes visible at the fistula ( Fig. 1 **b** ). Due to the small diameter of the fistula, suggesting potential for spontaneous healing, we placed a jejunal feeding tube and carefully monitored drainage output. However, after 20 days of conservative treatment, drainage remained copious and cloudy.

**Fig. 1:**
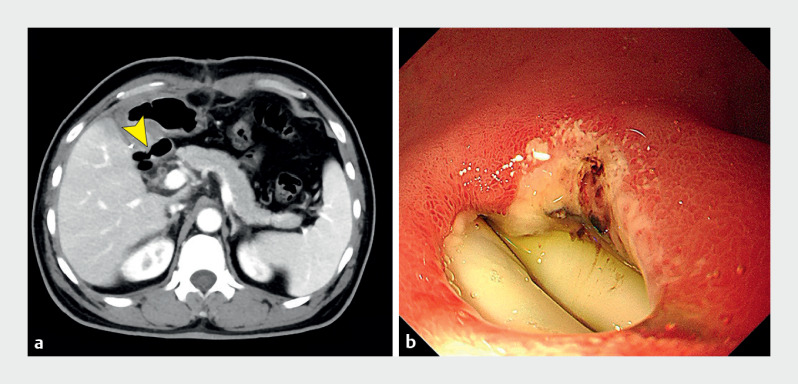
Examinations before endoscopic repair of a duodenal fistula. **a** Abdominal enhanced CT showed two abdominal drainage tubes in the right upper quadrant, with local thinning of the duodenal bulb wall closely adjacent to one of the drainage tubes (arrowhead). **b** Endoscopy demonstrated a 0.8-cm-diameter fistula in the duodenal bulb, and white drainage tubes were seen at the fistula.

Consequently, we performed endoscopic repair of the fistula ( Video 1 ). We observed an increased fistula size (3.0×2.0 cm) and employed 5 clips to achieve closure ( Fig. 2 ). Subsequent observations indicated a gradual reduction in abdominal drainage output and improved clarity. Follow-up examinations on postoperative day 20 revealed a residual clip and an ulcer on the duodenal wall, and no contrast medium outflow ( Fig. 3 ). Both tubes were subsequently removed, and the patient experienced a favorable recovery. Nine months postoperatively, endoscopy demonstrated smooth duodenal bulb mucosa with no ulcer.

Endoscopic repair of duodenal fistula occurring as a rare complication of abdominal drainage following partial hepatectomy.Video 1

**Fig. 2:**
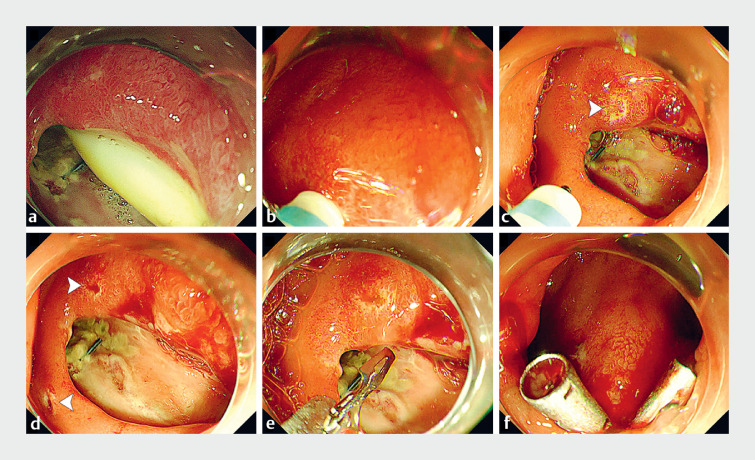
Procedure for endoscopic repair of the fistula. **a** During the procedure, we observed that the fistula size had increased to 3.0×2.0 cm. **b–d** The endoscopist created a mini hole on the edge on both sides of the wound using a dual knife (arrowheads). **e** The two jaws of the clip were inserted into the holes to achieve initial closure of the fistula. **f** In total, five clips were employed to complete final closure.

**Fig. 3:**
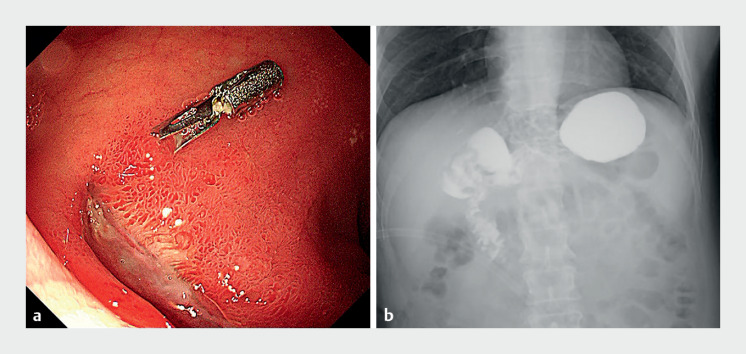
Follow-up examinations after endoscopic repair of the duodenal fistula. **a** Endoscopy on postoperative day 20 showed a residual clip and an ulcer on the duodenal wall. **b** Barium meal radiography on postoperative day 20 showed no contrast medium outflow.

Although duodenal fistulas or perforations related to abdominal drainage tubes are exceedingly rare, they can be life-threatening 3 . Our limited experience suggests that conservative treatment may not be sufficient for fistula healing, even in stable patients. Early identification and prompt endoscopic management of small duodenal fistulas could expedite patient recovery.

Endoscopy_UCTN_Code_TTT_1AQ_2AG

## Notes

Endoscopy E-Videos https://eref.thieme.de/e-videos
*E-Videos* is an open access online section of the journal *Endoscopy* , reporting on interesting cases and new techniques in gastroenterological endoscopy. All papers include a high-quality video and are published with a Creative Commons CC-BY license. Endoscopy E-Videos qualify for HINARI discounts and waivers and eligibility is automatically checked during the submission process. We grant 100% waivers to articles whose corresponding authors are based in Group A countries and 50% waivers to those who are based in Group B countries as classified by Research4Life (see: https://www.research4life.org/access/eligibility/ ). This section has its own submission website at https://mc.manuscriptcentral.com/e-videos .
